# Supplementary figures and images for: CoMiniGut—a small volume in vitro colon model for the screening of gut microbial fermentation processes
Source: PeerJ. 2018 Jan 19;6:e4268. doi: 10.7717/peerj.4268 (PMC5777374; doi:10.7717/peerj.4268)

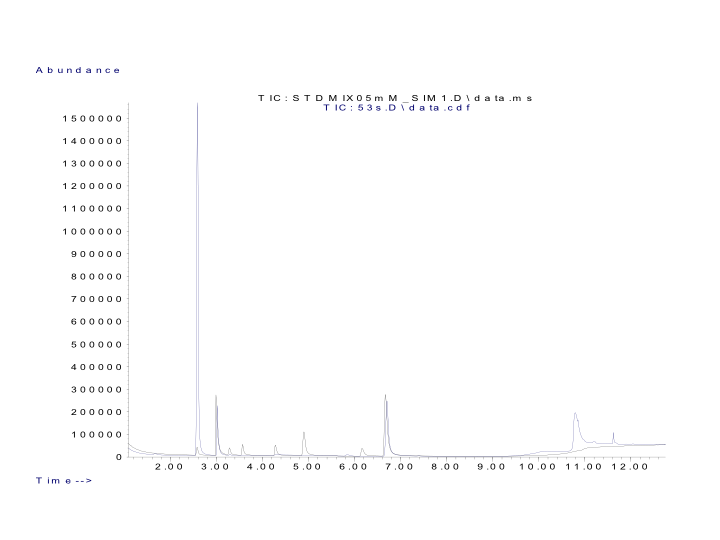

Supplement: Figure S1 — GC-MS chromatogram displaying the identified peaks (retention times in minutes): acetic acid (2.6), formic acid (3.0; product of oxalic acid), propionic acid (3.3), isobutyric acid (3.6), butyric acid (4.3), isovaleric acid (4.9) and 2-methyl-butyric (4.9; co-elute), valeric acid (6.2), 2 ethyl butyrate (6.7); overlaid with a sample chromatogram also showing the unknown peak at 5.85 min. [file peerj-06-4268-s001.png]

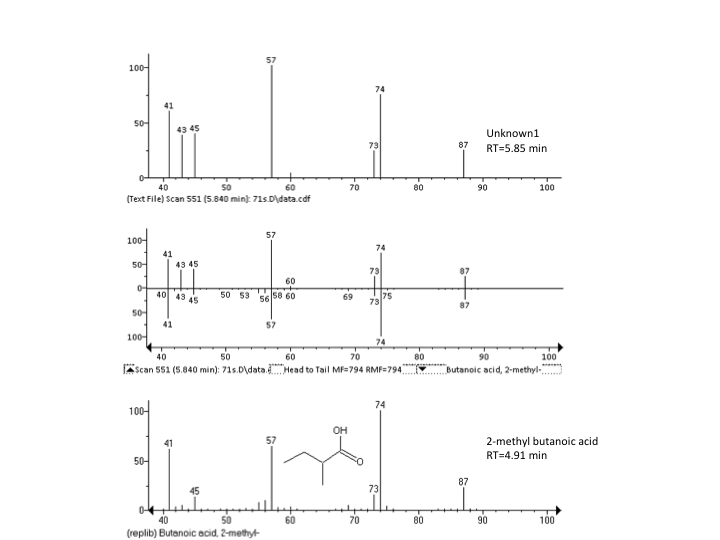

Supplement: Figure S2 [file peerj-06-4268-s002.png]

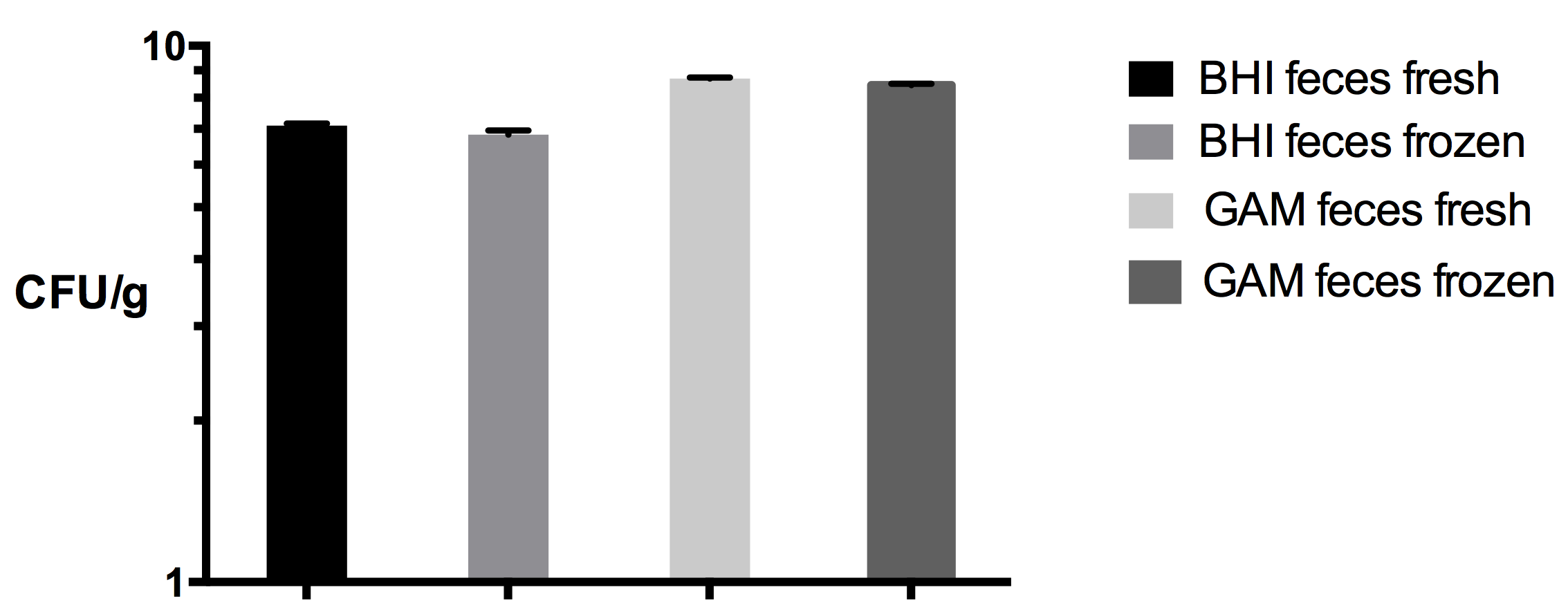

Supplement: Figure S3 — CFU/g counts of fresh and frozen fecal slurries on GAM and BHI media after anaerobic incubation for 72 h. No significant difference in the viability of bacteria from fresh and frozen fecal slurries was detected for individual weighted plate mean CFU/g (Students Paired T-test (p = 0.080). [file peerj-06-4268-s003.png]
